# Development of high-throughput methods to screen disease caused by *Rhizoctonia solani* AG 2-1 in oilseed rape

**DOI:** 10.1186/s13007-017-0195-1

**Published:** 2017-05-30

**Authors:** Fryni Drizou, Neil S. Graham, Toby J. A. Bruce, Rumiana V. Ray

**Affiliations:** 1 Division of Plant and Crop Sciences, School of Biosciences, University of Nottingham, Sutton Bonington Campus, Loughborough, Leicestershire UK; 2 School of Life Sciences, Keele University, Keele, Staffordshire UK

**Keywords:** *Rhizoctonia solani*, Oilseed rape, High-throughput phenotyping, Disease, Plant characteristics

## Abstract

**Background:**

*Rhizoctonia solani* (Kühn) is a soil-borne, necrotrophic fungus causing damping off, root rot and stem canker in many cultivated plants worldwide. Oilseed rape (OSR, *Brassica napus*) is the primary host for anastomosis group (AG) 2-1 of *R. solani* causing pre- and post-emergence damping-off resulting in death of seedlings and impaired crop establishment. Presently, there are no known resistant OSR genotypes and the main methods for disease control are fungicide seed treatments and cultural practices. The identification of sources of resistance for crop breeding is essential for sustainable management of the disease. However, a high-throughput, reliable screening method for resistance traits is required. The aim of this work was to develop a low cost, rapid screening method for disease phenotyping and identification of resistance traits.

**Results:**

Four growth systems were developed and tested: (1) nutrient media plates, (2) compost trays, (3) light expanded clay aggregate (LECA) trays, and (4) a hydroponic pouch and wick system. Seedlings were inoculated with virulent AG 2-1 to cause damping-off disease and grown for a period of 4–10 days. Visual disease assessments were carried out or disease was estimated through image analysis using ImageJ.

**Conclusion:**

Inoculation of LECA was the most suitable method for phenotyping disease caused by *R. solani* AG 2-1 as it enabled the detection of differences in disease severity among OSR genotypes within a short time period whilst allowing measurements to be conducted on whole plants. This system is expected to facilitate identification of resistant germplasm.

## Background

*Rhizoctonia solani* (Kühn) [teleomorph *Thanatephorus cucumeris* (Donk)] is a necrotrophic soil-borne fungus belonging to the phylum Basidiomycota. The species is sub-divided into anastomosis groups (AG) based on genetic and biological characteristics, as well as host-specific pathogenicity [1, 2]. Among the groups, AG 2-1 is the most destructive to oilseed rape (OSR, *Brassica napus*) and other members of the Brassicaceae [3, 4]. Under favourable temperatures, ranging from 18 to 20 °C, moist soil conditions and in the presence of the host, the growing hyphae infect young OSR seedlings causing pre- and post-emergence damping-off and root rot [4–6]. Damping-off is characterised by the formation of brown lesions and eventually rotting of the hypocotyl [7]. The infection can also result in root rot and stem rot in older plants [7, 8]. *B. napus* is a widely cultivated crop for oil production for human consumption and biodiesel, as well as for animal fodder. It is an amphiploid species derived from the crossing of *Brassica rapa* and *Brassica oleracea* and has undergone breeding for the optimisation of oil production and yields [9]. Although many studies have attempted to identify resistant or tolerant genotypes of *B. napus* and related species, currently there are no known resistant OSR genotypes to AG 2-1 [3, 5]. Babiker et al. [3] assessed the survival of 85 genotypes of *B. napus* and other *Brassica* species 4 weeks after sowing in inoculated soil. Their results showed that all genotypes were susceptible, the majority of seedlings died and only 18 genotypes survived with survival rates ranging from 8.3 to 88.3% [3].

The pathogen can be partially controlled using seed treatments prior to sowing [10] and via cultural practises [4, 8]. However, these control measures only reduce the inoculum in the soil and thus delay the infection. The use of biofumigation and seed meals, from Brassicaceous plants, that usually suppress soil-borne pathogens [11, 12] or the application of beneficial biological control organisms such as *Trichoderma* and binucleate *Rhizoctonia* [8], are not effective against *R. solani* AG 2-1. Consequently, the identification of traits and genes associated with resistance to *R. solani* AG 2-1 is an essential step towards the development of sustainable integrative control strategies for this pathogen.

An important factor in developing a method is to consider the epidemiology of the pathogen and the specificity of the pathosystem. In the case of *R. solani* and *B. napus* seed germination, emergence and survival under inoculated conditions can potentially reveal phenotypic differences among genotypes that play a role in susceptibility or resistance towards AG 2-1. The developmental rate of genotypes is likely to influence disease outcome [6, 8], therefore plants that emerge faster are expected to perform better. Additionally, plant characteristics such as hypocotyl length and root architecture may explain the ability of certain genotypes to escape infection. Furthermore, the progress of disease as well as its severity in different plant organs could potentially indicate genetic differences among different genotypes. At present the most popular method to assess disease severity and classify different genotypes and plant species to their susceptibility to *R. solani* is using pots with soil or soil-free media [3, 4, 7]. Although screening in soil is realistic and provides an ideal environment for the fungi, it is time consuming, labour intensive and requires extensive controlled environment space. This limits the number of plants that can be screened quickly and cheaply. Another major bottleneck in identification of resistance to soil-borne pathogens, apart from the time and space required when using inoculated soil or compost to cause disease, is the uncertainty and/or reproducibility of moderate disease on which to detect consistent differences between genotypes.

The aim of the present work was to develop a low cost, rapid and high-throughput method to enable the screening of OSR genotypes for identification of *R. solani* AG 2-1 resistance. Four different methods were tested: media nutrient plates, hydroponic growth in pouches and growth in trays with compost or light expanded clay aggregate (LECA). The methods were evaluated to screen disease and/or assess plant physiological characteristics within a short period of time during the early stages of infection among different OSR genotypes.

## Methods

### Inoculum and seeds

*Rhizoctonia solani* AG 2-1 (#1934 from the University of Nottingham isolate collection), originally isolated from OSR plants, was used to produce inoculum. The pathogenicity of this isolate to OSR was previously confirmed by Sturrock et al. [13]. The inoculum was grown on Potato Glucose Agar (PGA; Sigma-Aldrich, UK) at room temperature (18–20 °C) for a period of 10–14 days prior to the inoculation. In order to exclude contamination by other pathogens and ensure their germination, seeds were surface sterilised with 4% sodium hypochlorite (Parazone, Jeyes Limited, UK) for 5 min followed by three rinses with distilled autoclaved water and then pre-germinated on round filter paper (diameter 85 mm, GE Healthcare Whatman, UK) with 3 ml of sterile water and kept in dark at room temperature (18–20 °C) for 2 days. A group of eight *B. napus* genotypes, not previously tested for AG 2-1 resistance, was used for the evaluation of the methods to evaluate their performance against AG 2-1. The group consisted of seven commercial winter oilseed cultivars ‘Temple’(conventional), ‘Abaco’(conventional), ‘Lioness’(conventional), ‘Grizzly’(conventional), ‘Galileo’(conventional), ‘Sequoia’(semi-dwarf hybrid) and ‘ES Betty’(restored hybrid) and one fodder type (‘Canard’).

### Nutrient media plates

Square petri dishes-plates (120 × 120 × 17 mm Greiner Bio-One International) were filled with sterile 50% Hoagland No. 2 Basal Salt Mixture (Sigma-Aldrich, UK), pH 5.8 and 1% w/v agar (Agar–Agar granular powder, Fisher Scientific, UK). On each plate 3 seedlings of each genotype were placed 2 cm from the top of the plate with equal distances between them. For the inoculation, 1 plug (5 × 5 mm) of *R. solani* AG 2-1 from a colony growing on PGA was placed below each seed and 1 cm above the bottom of the plate. The control plates were not inoculated. Inoculated and control plates were sealed with parafilm and kept in an upright position in a controlled environment room at 18 °C and 12 h light:12 h dark. Photosynthetically active radiation (PAR) was 218.5 μmol s^−1^ m^−2^ at a height of 4 cm (LI-250A light meter, LI-COR Biosciences).

### Hydroponic growth in pouch and wick system

A method previously developed for high-throughput phenotyping of roots in tanks [14, 15] was modified for screening disease caused by *R. solani* AG 2-1. The construction of the tank consisted of a metal frame with 9 drip trays and 192 growth-pouch positions. Each pouch was made of an acrylic bar, onto which 2 filter papers (Anchor Paper Company, St Paul, MN, USA) were placed on each side and covered with a black polythene sheet (Cransford, Polyethylene Ltd, Suffolk, UK). The filter papers and the sheets were held on the bars with foldback clips (19 mm). Prior to sowing, pouches were left to soak overnight in nutrient solution (25% Hoagland’s in 2 L of purified water per tray). During the experiment filter papers on growth pouches remained soaked by adding purified water in the trays in equal amounts. Filter papers and clips were autoclaved and acrylic bars were bleached and sprayed with 70% ethanol prior to their use, to eliminate contamination. One seedling was placed in each side of the growth pouch, in the middle and approximately 3 cm from the top of the filter paper and left to grow for 3 days in a controlled environment room (18 °C, 12 h light:12 h dark). Then the seedlings were inoculated by adding 1 mycelia PGA plug (5 × 5 mm) 3 cm below the tip of the primary root and another 2 plugs diametrically opposite to each other and 3 cm away from the top of the primary root. For the control seedlings PGA plugs (5 × 5 mm) without inoculum were used.

### Growth in compost trays

Plastic trays (6143, Beekenkamp Verpakkingen, Netherlands) with 308 wells (3 × 3 cm) were filled with compost (Levington F2s, Everris Limited, UK) up to 2 cm and then each well was inoculated with 1 mycelia PGA plug (5 × 5 mm) of *R. solani* AG 2-1. A layer (0.5 cm) of compost was added above the inoculum and 3 pre-germinated surface sterilised seeds of OSR were placed in each well and covered with compost in order to fill up the well (1.5 cm layer). For the control wells 1 PGA plug without inoculum was added in each well. The trays were left in a controlled environment room (18 °C, 12 h light:12 h dark).

### Growth in light expanded clay aggregate (LECA) trays

Light expanded clay aggregate (LECA) was used to develop a screening method that kept the roots of young seedlings intact. Each compartment of a plastic tray (6143, Beekenkamp Verpakkingen, Netherlands) with 308 wells (3 × 3 cm) was filled with approximately 3 LECA particles (size 4–10 mm; Saint-Gobain Weber Limited, UK) enough to block the bottom and then 1 mycelia PGA plug (5 × 5 mm) of AG 2-1 was added for the inoculated treatment or 1 PGA plug for the control treatment. LECA particles were added to fill each compartment up to the ¾ of the well volume and then 2 pre-germinated seeds were added. Another layer of LECA was used to fill the wells to the top. An equal amount of 25% Hoagland’s in 0.5 L of purified water was supplemented in each well of the tray.

### Assessments on disease and plant characteristics

In nutrient media plates and in hydroponic pouches disease as well as plant characteristics (hypocotyl, primary root and lateral root length, lateral root and leaf number) were assessed using the same method but at different time points; Nutrient media plates were assessed at 4, 7 and 10 days post inoculation (dpi) while the seedlings in the hydroponic pouches only at 4 dpi. Disease assessment was made with disease severity categories modified from Khangura et al. [7]; for hypocotyl rot the seedlings were categorised on a scale of 0–3 (0 = no lesions, 1 = lesions on hypocotyls affecting <25% of the length of the hypocotyl, 2 = lesions covering 26–75% of the length of the hypocotyl, 3 = lesions covering >75% of the length of the hypocotyl), for primary root rot on a 0–6 scale (0 = no lesions, 1 = small lesions on primary root, 2 = discoloration up to 50% of primary root, 3 = discoloration 51–75% of the primary root, 4 = discoloration >75% and necrosis covering up to 30% of primary root, 5 = necrosis covering 31–60% of primary root, 6 = necrosis covering >61% or dead root) and for leaf disease on a 0–3 scale (0 = no lesions, 1 = disease affecting up to 25% of total leaf area, 2 = disease affecting 25–50% of total leaf area, 3 = disease affecting 51–75% of total leaf area, 4 = completely necrotic leaves of total leaf area). Disease index (DI %) was calculated as: [S (no. plants in disease category) × numerical value of disease category) × 100]/[(no. plants in all categories) × (maximum value on rating scale)]. Plant images were taken from the plates using a digital SLR camera (Canon 1100D, EOS Utility software, Canon Inc., Tokyo, Japan) and analysed with ImageJ (version 1.4.7, Schneider et al. [16]) software and used for the assessment of plant characteristics.

In compost trays, emergence and survival were assessed daily, 2 days after planting and for a period of 5 days. Final counts of emergence and survival were taken on the 10 dpi and then seedlings were removed from the wells, washed and assessed for disease. For non-emerged seedlings, soil was removed and examined to ensure that control seedlings (or seeds) were healthy while the inoculated were heavily infected (dead). For the disease assessments, the above disease scale was modified by including another level for seedlings suffering from pre-emergence damping-off (not emerged) and those that they did not survive due to post-emergence damping-off. Thus for hypocotyl rot, seedlings were rated on a 0–4 scale (4 = completely dead or/and not emerged), for primary root rot on a 0–7 scale (7 = completely dead or/and not emerged) and for leaf disease on a 0–5 scale (5 = not emerged). The percentage of disease index was calculated as described before. Control seedlings that did not emerge were scaled as healthy, as they were found in the compost without any disease symptoms.

Survival of seedlings in trays with LECA was estimated 5 dpi, then the seedlings were removed and images were taken to estimate disease (Canon 1300D, EOS Utility software, Canon Inc., Tokyo, Japan) and analysed with ImageJ (version 1.4.7, software). Seedlings that had not emerged in the control treatment, were assessed in order to ensure that they were viable and not infected, contrary with seedlings that had not emerged in the inoculated treatment which were heavily infected. In contrast to the other methods, disease was estimated as a percentage of the infected plant area to the total plant area for hypocotyls and for roots.

### Experimental design and statistical analysis

All statistical analysis was performed using GenStat (15th Edition, VSN International Ltd, Hemel Hempstead, UK). The experiments for each method were designed as randomized blocks with two factors; genotype and inoculum. Where appropriate disease development, seedling emergence, survival and plant characteristics were analysed using analysis of variance (ANOVA) for repeated measures. General ANOVA was used for variables assessed less than three times. Each method consisted of two replicated experiments, analysed as replicates when there were no significant interactions detected. Disease progress on the genotypes was analysed by excluding the non-inoculated controls in each of the four methods.

## Results

### Nutrient media plates

**Fig. 1:**
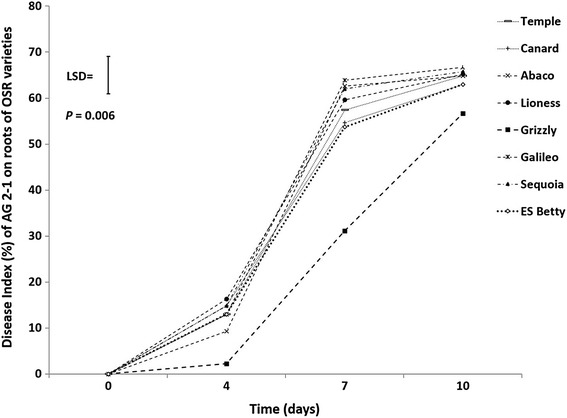
Progress of disease caused by AG 2-1 on roots of seedlings of the eight varieties growing in media plates

Disease development on the roots of inoculated seedlings in nutrient media plates revealed significant differences during the 10 days of the experiment (*P* = 0.006; Fig. 1). Disease developed slower on the genotype ‘Grizzly’, which had consistently less disease compared to the other genotypes. ‘Abaco’ followed ‘Grizzly’ but did not have significantly different disease severity compared to the other genotypes (Fig. 1). Disease on hypocotyl and roots was inconsistent between the two replicate experiments (results not shown).

Over time, AG 2-1 significantly reduced the length or the number of assessed plant characteristics apart from hypocotyl length (Table 1). Inoculated seedlings had significantly fewer leaves, smaller and fewer lateral roots, shorter primary roots and as a result total length of roots was also reduced (Table 1). However, hypocotyl growth was not different between inoculated and control seedlings (*P* = 0.216). There were no interactions between inoculum and genotype and in both inoculated and un-inoculated seedlings consistent differences were observed in the growth of each of these plant characteristic between the different days (Table 2). Lateral root length (*P* < 0.001) and total root length (*P* = 0.001) were significantly different between the different genotypes over the 10 days. Hypocotyl length was different among the varieties for each of the 3 days, with ‘Grizzly’ always having shorter hypocotyl and longest lateral roots (Table 2). Additionally, the number of lateral roots was also significantly different between the genotypes with ‘Canard’ always having more lateral roots. Significant differences for primary root length between varieties were observed for day 4 and 7 but not on day 10. Seedlings of ‘Grizzly’ had consistently shorter primary roots (Table 2). Significant differences between genotypes in total length of the roots and number of leaves were observed only on the 4th and 7th day, respectively.

**Table 1:** Plant characteristics under inoculated (AG 2-1) and un-inoculated (control) conditions during the 10 days of the experiment in nutrient media plates

Treatment	Hypocotyl length			Leaves number			Lateral RL			Lateral root number			Primary RL			Total RL		
	4 days	7 days	10 days	4 days	7 days	10 days	4 days	7 days	10 days	4 days	7 days	10 days	4 days	7 days	10 days	4 days	7 days	10 days
AG 2-1	1.99	2.30	2.37	2	3.14	1.97	1.27	2.26	2.63	12.21	21.64	23.1	7.09	7.57	7.48	8.37	9.83	10.07
Control	1.78	2.06	2.37	1.99	3.12	3.84	1.16	2.37	3.30	10.79	22.42	26.72	7.98	10.60	11.51	9.13	12.97	14.81
*P*_(time*inoculum)_	0.216			<0.001			<0.001			0.021			<0.001			<0.001		
LSD_(time*inoculum)_	0.26			0.23			0.44			3.15			1.07			1.20		
*P*_(time)_	<0.001			<0.001			<0.001			<0.001			<0.001			<0.001		
LSD_(time)_	0.15			0.15			0.18			1.76			0.30			0.35		

Lengths are expressed as cm. *P*_(time*inoculum)_ values and LSD_(time*inoculum)_ (ANOVA) were used for the comparison between the two treatments and *P*_(time)_ values and LSD_(time)_ for the comparison among different days

*RL* root lengths

**Table 2:** Plant characteristics of the tested genotypes, in nutrient media plates

Genotype	Hypocotyl length			Leave number			Lateral RL			Lateral root number			Primary RL			Total RL		
	4 days	7 days	10 days	4 days	7 days	10 days	4 days	7 days	10 days	4 days	7 days	10 days	4 days	7 days	10 days	4 days	7 days	10 days
Temple	2.25	2.34	2.87	2.00	3.25	3.14	1.11	1.61	2.76	9.28	18.94	20.92	7.59	9.46	9.03	8.71	11.06	11.79
Canard	2.11	2.68	2.81	2.00	3.61	3.06	1.34	2.23	2.87	18.19	29.31	30.31	8.79	10.22	10.33	10.13	12.45	13.20
Abaco	1.77	2.08	2.10	2.01	3.24	3.07	1.30	2.09	2.53	14.12	22.70	26.67	6.83	8.19	8.93	8.14	10.28	11.45
Lioness	2.23	1.27	1.35	2.00	3.18	2.81	1.25	2.25	2.72	12.49	20.89	23.65	7.68	8.82	9.23	8.93	11.06	11.95
Grizzly	1.18	1.27	1.35	2.00	2.97	3.07	1.16	3.11	4.23	6.71	18.32	24.00	4.65	7.16	7.92	5.81	10.26	12.16
Galileo	1.73	1.99	2.27	2.00	2.78	2.49	1.04	2.34	2.76	11.78	25.00	29.94	8.08	9.91	10.43	9.12	12.24	13.19
Sequoia	1.79	2.01	2.03	2.00	3.14	2.93	1.29	2.38	3.14	10.75	18.14	23.03	8.91	9.72	10.08	10.20	12.10	13.22
ES Betty	1.99	2.31	2.52	2.00	2.86	2.69	1.23	2.51	2.73	8.69	22.97	20.78	7.74	9.22	9.85	8.97	11.73	12.58
*P*	<0.001	<0.001	<0.001	0.99	<0.001	0.254	0.781	0.041	0.043	<0.001	0.105	0.001	<0.001	0.021	0.186	<0.001	0.129	0.49
LSD	0.39	0.39	0.59	0.07	0.32	0.57	0.41	1.15	1.02	3.26	8.04	5.35	1.35	1.59	1.86	1.40	1.57	1.88

Lengths are expressed in cm. Comparisons for each plant characteristic among genotypes were made by using *P* values and LSD (ANOVA)

*RL* root length

### Hydroponic growth in pouch and wick system

Infection of seedlings with AG 2-1 did not result in significant differences in disease severity between the genotypes for any of the examined plant organs (Table 3). Inoculated seedling characteristics were all significantly affected by disease 4 dpi compared to their controls except for lateral root number (*P* = 0.066; Table 4). Additionally, significant variation was observed between genotypes for some of their morphological characteristics (Table 4): hypocotyl length (*P* < 0.001), lateral root length (*P* = 0.011) and lateral root number (*P* = 0.011) were significantly different. The length of the hypocotyl was significantly reduced in infected seedlings with ‘Grizzly’, ‘Galileo’ and ‘Sequoia’ being most affected. ‘Canard’ had the least reduction and ‘ES Betty’ had no reduction in hypocotyl length despite the disease (Table 4). In general, ‘Canard’ had shorter hypocotyls compared to the rest while ‘Abaco’ and ‘Sequoia’ had longer ones. The number of leaves of inoculated seedlings was significantly reduced compared to controls for all genotypes but no differences were observed among the genotypes. Lateral roots of genotypes were significantly shorter under inoculation with ‘ES Betty’ and ‘Grizzly’ being more affected with reduction of length of 72.2 and 88.1% respectively. Although lateral root length was significantly reduced in infected seedlings, lateral root number was not affected. Nevertheless, genotypes differed in the number of lateral roots with ‘Canard’ having more lateral roots. The length of the primary roots was significantly reduced due to infection of AG 2-1 in all genotypes with more pronounced reduction in ‘Grizzly’ (61.8%), ‘Sequoia’ (55.9%) and ‘ES Betty’ (48.5%). The total length of roots was also significantly reduced due to the infection with AG 2-1 with ‘ES Betty’, ‘Sequoia’ and ‘Grizzly’ having the greatest reduction of length. Despite the effect of AG 2-1 infection the genotypes did not significantly differ in primary and total root lengths (Table 4).

**Table 3:** Disease index on hypocotyls, roots and leaves of the tested genotypes after inoculation with AG 2-1 for 4 days on the hydroponic growth pouches

Genotype	Disease index (%)		
	Hypocotyl	Root	Leaves
Temple	61.1	54.2	22.9
Canard	69.4	68.1	35.4
Abaco	66.7	54.2	18.8
Lioness	69.4	54.2	18.8
Grizzly	72.2	72.2	47.9
Galileo	77.8	45.8	37.5
Sequoia	75.0	52.8	35.4
ES Betty	58.3	58.3	16.7
*P*	0.935	0.663	0.533
LSD	32.88	29.28	34.88

For the comparison of disease severity among genotypes within each plant part *P* values and LSD were used (ANOVA)

**Table 4:** Comparison of plant characteristics between inoculated (AG 2-1) and un-inoculated (Control) seedlings of different OSR genotypes 4 days after inoculation on hydroponic growth pouches

Genotype	Hypocotyl length		Leaves number		Lateral RL		Lateral root number		Primary RL		Total RL	
	AG 2-1	Control	AG 2-1	Control	AG 2-1	Control	AG 2-1	Control	AG 2-1	Control	AG 2-1	Control
Temple	1.87	2.19	1.63	1.99	0.52	1.29	2.00	4.85	1.40	2.44	1.92	3.73
Canard	1.31	1.40	1.50	2.08	1.15	1.62	4.67	5.83	2.09	2.83	3.24	4.45
Abaco	2.79	2.93	2.00	2.00	0.49	0.91	2.33	3.17	1.85	2.40	2.34	3.30
Lioness	2.13	2.62	1.33	2.08	0.54	0.93	3.92	3.58	1.94	2.98	2.48	3.91
Grizzly	1.48	2.41	1.46	1.99	0.15	1.22	0.42	1.65	0.68	1.78	0.82	3.00
Galileo	1.81	2.54	1.54	2.00	0.32	0.49	1.33	1.08	1.39	2.55	1.70	3.04
Sequoia	2.50	3.16	1.54	2.00	0.33	1.18	2.33	3.25	1.50	3.40	1.83	4.58
ES Betty	2.50	2.34	1.71	2.00	0.47	1.69	2.75	5.08	1.74	3.38	2.21	5.07
*P*_(genotype)_	<0.001		0.721		0.011		0.011		0.299		0.115	
LSD_(genotype)_	0.56		0.32		0.49		2.34		1.13		1.37	
*P*_(inoculum)_	0.005		<0.001		<0.001		0.066		<0.001		<0.001	
LSD_(inoculum)_	0.28		0.16		0.25		1.17		0.57		0.68	

Lengths are expressed in cm. *P*_(genotype)_ and LSD_(genotype)_ were used for the comparison among genotypes and *P*_(inoculum)_ and LSD_(inoculum)_ for the comparison between treatments (ANOVA)

*RL* root length

### Growth in compost trays

**Fig. 2:**
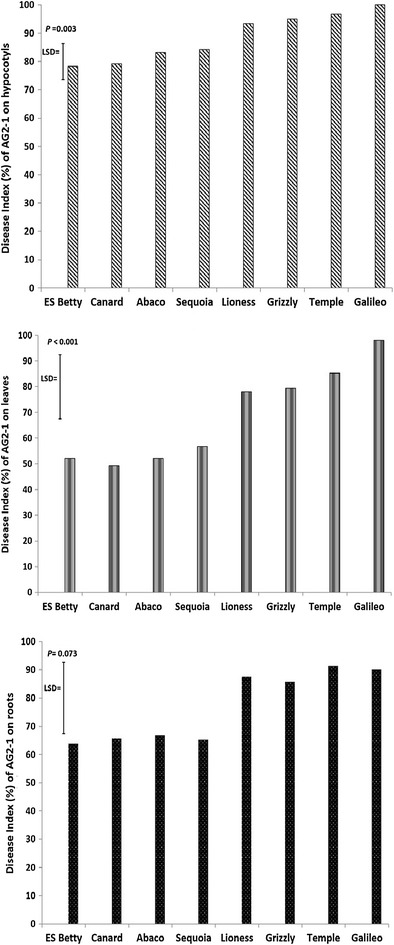
Disease on hypocotyls, leaves and roots of the tested genotypes 10 days after inoculation in compost trays

Inoculation of seedlings in compost trays with AG 2-1 resulted in significant differences on disease severity between the genotypes on hypocotyls (*P* = 0.003) and leaves (*P* < 0.001) but not in roots (*P* = 0.073; Fig. 2). ‘ES Betty’ and ‘Canard’ were consistently least affected, followed by ‘Abaco’ and ‘Sequoia’, ‘Lioness’ and ‘Grizzly’ (Fig. 2). ‘Galileo’ and ‘Temple’ were the genotypes with significantly more disease (Fig. 2).

**Fig. 3:**
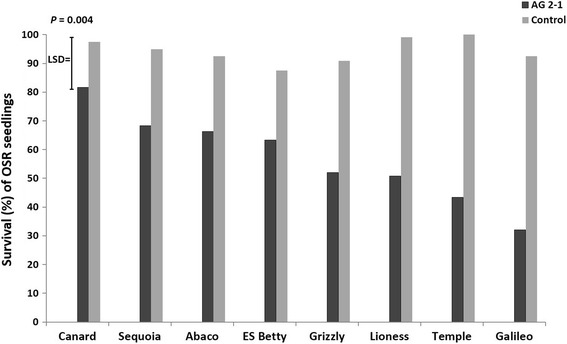
Percentage of survival of different OSR genotypes 10 days post inoculation in compost trays. Comparisons for the interaction between treatment and genotype were made with *P* values and LSD (ANOVA)

Emergence of seedlings was significantly different between genotypes (*P* < 0.001) and inoculation with AG 2-1 reduced seedling emergence in almost all varieties apart from ‘Canard’, ‘Grizzly’ and ‘ES Betty’ (*P* < 0.001). However, there was no significant interaction between genotypes and treatment (*P* = 0.186) (Table 5). Infection of seedlings with AG 2-1 enabled us to detect differences in survival between inoculated and non-inoculated control seedlings (*P* < 0.001) and there were significant differences between genotypes in seedling survival (*P* = 0.004; Fig. 3). ‘Canard’ was the genotype with significantly greater survival and the only one with no significant differences between inoculated and control seedlings (Fig. 3). ‘Sequoia’, ‘Abaco’, ‘ES Betty’ and ‘Grizzly’ followed, with the first two not being significantly different from ‘Canard’. The poorest survival was observed for ‘Galileo’, ‘Temple’ and ‘Lioness’ (Fig. 3).

**Table 5:** Comparison of emergence between inoculated (AG 2-1) and un-inoculated (Control) seedlings of different OSR genotypes 10 dpi in compost trays

Genotype	Emergence (%)	
	AG 2-1	Control
Temple	30.0	67.2
Canard	83.9	88.3
Abaco	60.0	98.3
Lioness	43.3	88.9
Grizzly	42.8	57.8
Galileo	11.7	64.4
Sequoia	63.3	98.9
ES Betty	53.3	77.2
*P*_(genotype)_	<0.001	
LSD_(genotype)_	18.586	
*P*_(inoculum)_	<0.001	
LSD_(inoculum)_	9.293	
*P*_(inoculum*genotype)_	0.186	
LSD_(inoculum*genotype)_	26.284	

*P*_(inoculum)_ and LSD_(inoculum)_ were used for the comparison between treatments and *P*_(inoculum*genotype)_ and LSD_(inoculum*genotype)_ for the interaction between genotypes and treatments (ANOVA)

### Growth in LECA trays

**Fig. 4:**
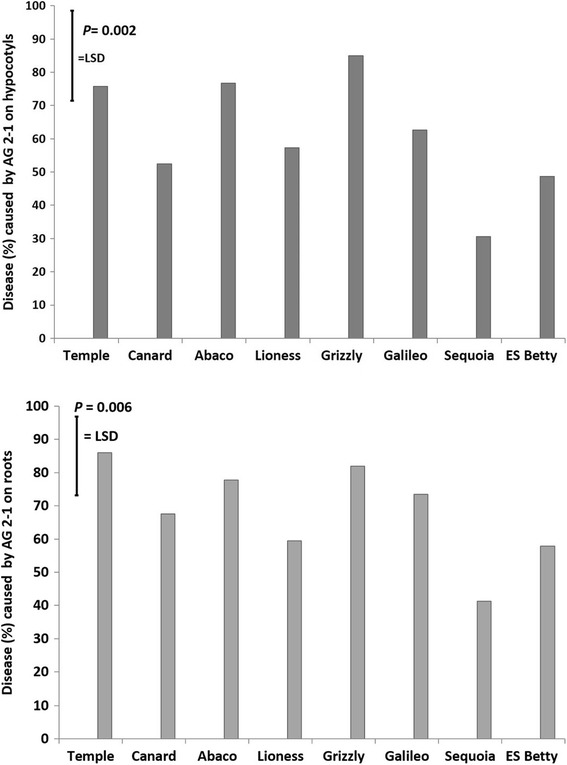
Disease on hypocotyls and roots of the tested genotypes 5 days after inoculation in trays with LECA

AG 2-1 was able to grow and infect seedlings grown in trays filled with LECA. The inoculation resulted in disease symptoms 5 days post inoculation (*P* < 0.001) and enabled assessment through image analysis. Screening for disease revealed significant differences between the tested genotypes for both disease on hypocotyls (*P* = 0.002) and on roots (*P* = 0.006). ‘Sequoia’ was the genotype with consistently less disease on both roots and hypocotyls followed by ‘ES Betty’ (Fig. 4). ‘Canard’ and ‘Lioness’ ranked in the middle and had significantly lower disease than ‘Grizzly’ (*P* = 0.002). ‘Galileo’, ‘Temple’, ‘Abaco’ and ‘Grizzly’ were the genotypes with the highest disease levels (Fig. 4). Disease severity on roots indicated that genotypes had similar responses to AG 2-1 infection: ‘Sequoia’ was the genotype with the least disease followed by ‘ES Betty’ and ‘Lioness’; ‘Canard’ ranked in the middle, and ‘Temple’ was the genotype with the most severe disease symptoms on roots (*P* = 0.006; Fig. 4).

Inoculation with AG 2-1 reduced seedling survival (*P* < 0.001) 5 dpi but survival was not significantly different between genotypes (*P* = 0.107) and no significant interaction was observed between genotypes and treatment (*P* = 0.716).

## Discussion

The primary aim of this study was to develop a high throughput method for evaluation of OSR resistance to disease caused by *R. solani* AG 2-1, as a first step towards the identification of traits that could be used in future breeding programs. Early infection of OSR by *R. solani* AG 2-1 leads to pre- and post-emergence damping off which reduces crop establishment, but infection in later stages towards the maturity of plant is less damaging [8]. Therefore, our objective was to develop methods to enable assessment of the early stages of disease progression. A key aspect of our work was to develop a low cost, rapid method that would enable screening of a large number of different OSR genotypes. The four developed methods here (nutrient media plates, hydroponic growth in pouches, trays with compost or LECA) lasted no more than 10 days and enabled the screening of up to 240 seedlings. We used a simple and cheap inoculation technique with mycelial plugs, which allows the induction of disease symptoms and minimises the time for inoculum production to 7 days.

Plant growth in media plates is a commonly used method for the evaluation of seedling growth and root architecture phenotyping. We aimed to further test this for the assessment of initial infection and disease development. Our results indicated that nutrient media plates are a good method for disease phenotyping of roots: both fungal hyphae and root systems grew successfully on the surface of the media. All the steps of infection and disease development could be observed and differences in disease severity amongst different genotypes were detected. Also, due to the horizontal growth of the root system, root architecture was easily measured. Unfortunately, in contrast to roots, this method is not suitable for assessing disease in hypocotyls and leaves. There was no consistency in disease severity among genotypes between the two replicate experiments with hypocotyls. In many cases, hypocotyls escaped hyphae and tended to grow towards the lids of the plates. In the same way the leaves of these plants were also escaping the pathogen. Consequently, this variation in growth led to the uneven and inconsistent infection among genotypes and between experiments. Nonetheless, disease significantly affected both leaves and roots of inoculated seedlings compared to controls, with reduction of healthy leaf area, root length (both primary and lateral) and lateral root number. The results are in agreement with a recent study showing that AG 2-1 causes severe disease by significantly reducing root length and density of inoculated OSR plants and is capable of killing the seedling within 6 dpi [13]. The analysis of plant characteristics showed that genotypes differ in lateral root and total root length as well as their growth rates. Among the genotypes, ‘Grizzly’ was the only one that consistently had significantly lower disease but also shorter hypocotyl and primary root compared to other genotypes. Therefore, it might be that the slower growth rate contributed to delay in infection and thus resulted in lower disease levels observed on plates. ‘Grizzly’ is a winter hybrid known to carry genes for stem canker resistance and for that reason is included in breeding programs [17], however in our tests with 56.6% of root disease ‘Grizzly’ was susceptible to AG 2-1.

Advanced high-throughput methods have been developed to screen the root system [18] and to quantify traits and identify Quantitative Trait Loci (QTLs) [14]. Atkinson et al. [14] screened a mapping population of wheat seedlings aiming to identify QTLs linked with root traits in hydroponic pouch and wick system. Also Thomas et al. [15] used this approach for screening a range of OSR genotypes under control environment and field conditions. Here we modified the method for screening disease caused by AG 2-1 in OSR. Our results showed that *R. solani* was able to grow on filter paper and infect young OSR seedlings causing disease symptoms 4 dpi. Within this time, disease developed on hypocotyls, roots and leaves and resulted in their reduction in inoculated plants compared to controls. However, no differences were detected between genotypes for disease and all were observed to be highly susceptible under this method of inoculation. It is likely that the tested genotypes are characterized by only minor differences and the present screening method could not detect them under the tested conditions. However, this is in contrast with the results of the other two methods, where significant differences on disease severity were observed. Different inoculum densities and length of inoculation periods were tested (results not shown) prior to the present experimental procedure, which appeared to be the most consistent. Possibly the moist environment of the filter paper and the polythene sheet as well as the lack of the soil environment altered hyphal growth and the infection process. *R. solani* is a soil-borne pathogen, thus the presence of soil with nutrients, organic matter and aeration play a pivotal role in its epidemiology. In this growing system the polythene sheet was attached to the filter paper but in the position of the seedlings, small aerate cavities were formed possibly enabling the pathogen to grow better. As a result, pathogen hyphae were denser close to the seedling and eventually led to greater disease on plants, whilst in the other methods pathogen growth was more even. Nonetheless, this method enabled us to detect differences in plant characteristics between inoculated and un-inoculated control seedlings, as well as differences among genotypes in a short period of time.

Soil and compost are most commonly used for the evaluation of plant resistance against soil-borne pathogens. In the case of *R. solani*, the vast majority of studies focussing on plant responses to pathogen exposure, have used soil [3, 5], soil free media [4] or a combination of both [10]. In this way, the experiments simulate more realistic conditions that occur in the field and a better evaluation of the plants response to the pathogen is observed. Therefore we decided as a suitable alternative that the third method should be developed with compost. In contrast to other studies, we used multiple cell-trays which save space and time by enabling us to screen more than 100 different genotypes per tray in a single experiment. The trays were also ideal to assess the early stages of infection in young seedlings that are less than 10 days old. An additional benefit of this method is that it enabled the recording of emergence and survival of seedlings and hence record pre- and post-emergence damping off. Low emergence of inoculated seedlings compared to controls, indicated susceptibility of those cultivars to pre-emergence damping off and confirmed the detrimental effect of AG 2-1 to OSR during early growth stages.

Disease screening on hypocotyls and leaves was easily conducted, but in contrast the extraction and assessment of the delicate roots of seedlings damaged by root rot was difficult and time consuming. Despite meticulous work, it was hard to keep the roots intact. We were unable to detect significant differences in root disease between cultivars in this method but we were able to detect differences in disease severity of hypocotyls and leaves. ‘ES Betty’ and ‘Canard’ were consistently the two genotypes with the lowest disease while ‘Temple’ and ‘Galileo’ were the most susceptible. This is in agreement with emergence and survival data and it can be an indication that these genotypes may carry both quantitative and qualitative traits allowing them to perform better against AG 2-1. In this research all genotypes were pre-germinated in order to standardise our methods, and therefore their germination rates under inoculated conditions were not assessed. However, it is possible that some genotypes are able to germinate and emerge faster and therefore escape and/or be less affected by the infection. Indeed, Sturrock et al. [13], suggested that rapid germination of OSR seedlings may enable the early establishment of a strong root system allowing better nutrient uptake and growth and consequent recovery from AG 2-1 infection.

We aimed to improve the method by eliminating high inoculum pressure and most importantly by reducing damage to roots to be able to better discriminate the genotypes in our disease assessments. Therefore we decided first to reduce the time that the seedlings were exposed to the pathogen from 10 to 5 dpi. Secondly we used a medium that would not affect seedling growth but would minimise the damage to the root system upon removal. In this respect, LECA particles with the addition of nutrient solution appeared to be an appropriate medium. LECA has been receiving a growing acceptance as an environmental friendly natural material with great benefits in civil engineering and gardening. Currently there is a limited number of published studies examining the use of LECA as a growing medium [19–21] and to the best of our knowledge only one study has examined the growth of a fungi in LECA [22]. In this study the authors showed that arbuscular mycorrhizal fungi (AMF) were not able to colonise their tested plant, *Paspalum notatum*, when grown in LECA and consequently concluded that LECA was not colonised effectively by AMF [22]. However, the results of the current study show that the necrotrophic pathogen *R. solani* AG 2-1 was able to grow on the surface of LECA particles, observed as hyphal mass and infect OSR seedlings. The inoculation period of 5 days was sufficient to induce disease symptoms without killing the seedlings. At the same time differences in disease severity of the tested genotypes were detected for both hypocotyls and roots. The use of LECA preserved the roots intact during their collection from the trays and therefore allowed more accurate disease assessments. Taking images of the seedlings and analysing them with ImageJ not only allowed us to complete the experiments faster but also to estimate the disease more objectively compared to more subjective visual assessments which are not taking into account differences in growth and development of the seedlings. The OSR genotypes had different responses to AG 2-1 infection: ‘Sequoia’ was the least affected for both damping off and root rot, followed by ‘ES Betty’. Although disease affected the survival of inoculated OSR seedlings compared to the controls, we were not able to detect significant differences in survival of seedlings between the different genotypes at 5 dpi.

### Comparison of different methods

Assessing the severity of disease caused by AG 2-1 on hypocotyls and/or roots of young seedlings is the most important measure for the identification of active genetic resistance. Nonetheless, other traits related to rapid development and growth for crop establishment such as root architecture and emergence or survival are important for the identification of disease escape. Each of the four methods we developed has positive and negative aspects: Nutrient media plates enabled the recording of the infection progress and the collection of data on root traits but were not suitable for disease screening of hypocotyls and leaves. Growth in hydroponic pouches can be high-throughput, fast screening method but the moist environment altered *R. solani* growth and we could not detect any difference in disease severity among the tested OSR genotypes. Screening on trays with compost was more realistic approach that makes available holistic disease screens for the plant as well as measurements of emergence and survival. Nevertheless, damage to the root system prevented accurate disease assessment and measurements of root architecture traits and a longer time was required to detect differences. However, the use of LECA holds the benefits of screening in compost trays but also enables the roots to be intact and detect differences between genotypes in root rot disease. We were unable to detect differences in survival most likely due to short infection period of 5 dpi. Most importantly 5 dpi screening in LECA resulted in moderate disease of seedlings compared to screening in compost and this might be the reason that we have small differences in in the ranking of genotypes between the two methods. Considering the severity of disease 5 dpi and the lack of resistance in the tested genotypes, further screening for a longer period for detection of differences in survival using this method was not pursued here. In Table 6 we provide a basic estimation of the cost of screening 100 genotypes by each method, based on the cost of consumables and equipment used; the hydroponic pouch and wick system was the most expensive method as the requirements for building the system were high compared to the other methods that use petri dishes and well trays. As mentioned previously, the choice of method should be based on the scientific aim; in the present study we aimed to identify a low cost high-throughput screening method which would enable the detection of potential resistant OSR genotypes to root diseases such as AG 2-1. Therefore, we required a method that allowed the detection of differences in disease severity and resultant changes to plant morphological characteristics. Screening in trays with LECA fulfilled these criteria it enables fast and high-throughput screening with the assessment of early infection stages. Therefore it is an applicable method for the detection of resistant OSR cultivars to AG 2-1.

**Table 6:** Estimation of cost for the screen of 100 genotypes in the developed methods

Method	Cost (£) for 100 genotypes
Hydroponic pouch and wick system	348
Nutrient media plates	27.3
Trays with compost	1.05
Trays with LECA	2.14

The estimation excludes the cost for the camera that was used in the hydroponic pouch and wick system, on nutrient media plates and trays with LECA

## Conclusion

The present study provides a new low cost, high-throughput screening method for the identification of potential OSR cultivars that are resistant to root diseases such as *R. solani* AG 2-1. This method can be used as an early step for the evaluation of germplasm prior to testing under field conditions. Additionally, it confirms that AG 2-1 is an extremely pathogenic isolate to OSR [3–6]; the inoculum density used resulted in low survival of young seedlings 10 dpi in compost trays and high disease levels ranked from 30 to 85% 5 dpi in trays with LECA. None of the genotypes tested in the current study were resistant. Future screening of diverse populations of *B. napus* and *Brassica* “species is essential to elucidate if there is any resistance against this destructive pathogen.

## Abbreviations

OSR: oilseed rape
AG: anastomosis group
LECA: light expanded clay aggregate
PGA: potato glucose agar
dpi: days post inoculation
DI: disease index
LSD: least significant difference of means
RL: root length
QTL: quantitative trait loci
AMF: arbuscular mycorrhizal fungi
